# Retraction: Application of a Mathematical Model in Determining the Spread of the Rabies Virus: Simulation Study

**DOI:** 10.2196/98666

**Published:** 2026-04-28

**Authors:** JMIR Publications Editorial Office

**Affiliations:** 1 JMIR Publications Toronto, ON

The JMIR Publications Editorial Office is retracting the article:

Application of a Mathematical Model in Determining the Spread of the Rabies Virus: Simulation Study [1]

This action is being taken due to identified concerns regarding potential manipulation of the submission process and authorship, the integrity of the peer review process, and the relevance of several references.

The concerns with this publication were identified during an investigation of a series of articles with related concerns. In response to our request for comment, author Yihao Huang indicated that the identified references were cited in a broader methodological context on computational modeling. However, as these references supported statements related to the history and transmission of rabies, the JMIR Publications Editorial Office determined that this response did not fully resolve the concerns regarding the initial use of these references in the published article.

Yihao Huang disagreed with the retraction. Author Mingtao Li did not reply directly and could not be reached regarding the decision to retract the article.
